# Zika Virus Infection and Guillain–Barré Syndrome in Three Patients from Suriname

**DOI:** 10.3389/fneur.2016.00233

**Published:** 2016-12-22

**Authors:** Thomas Langerak, Harvey Yang, Mark Baptista, Laura Doornekamp, Tessa Kerkman, John Codrington, Jimmy Roosblad, Stephen G. S. Vreden, Erwin De Bruin, Ramona Mögling, Bart C. Jacobs, Suzan D. Pas, Corine H. GeurtsvanKessel, Chantal B. E. M. Reusken, Marion P. Koopmans, Eric C. M. Van Gorp, Henk Alberga

**Affiliations:** ^1^Department of Viroscience, Erasmus Medical Center, Rotterdam, Netherlands; ^2^Department of Neurology, Academic Hospital Paramaribo, Paramaribo, Suriname; ^3^Department of Neurology, St. Vincentius Hospital, Paramaribo, Suriname; ^4^Diagnostic Laboratory, Academic Hospital Paramaribo, Paramaribo, Suriname; ^5^Department of Internal Medicine, Academic Hospital Paramaribo, Paramaribo, Suriname; ^6^Department of Neurology, Erasmus Medical Center, Rotterdam, Netherlands; ^7^Department of Immunology, Erasmus Medical Center, Rotterdam, Netherlands

**Keywords:** Zika virus, Guillain–Barré syndrome, *Flavivirus* infections, acute flaccid paralysis, emerging infectious disease

## Abstract

We present three patients from Suriname who were diagnosed with Guillain–Barré syndrome (GBS) during the Zika virus (ZIKV) outbreak in this country. One patient had a positive ZIKV urine real-time RT-PCR (qRT-PCR) result. The other two patients had a negative ZIKV urine qRT-PCR but a positive virus neutralization test and presence of IgG antibodies against ZIKV in the serum. Considering the evidence of a past ZIKV infection and absence of evidence for recent infections with the most common preceding infections of GBS, it is very likely that these GBS cases were triggered by ZIKV.

## Introduction

Since the Zika virus (ZIKV) outbreak in South America, an increased incidence of microcephaly and the Guillain–Barré syndrome (GBS) is reported in several affected countries in South America (1, 2).

Guillain–Barré syndrome is a heterogeneous postinfectious immune-mediated syndrome characterized by rapidly progressive symmetrical muscle weakness and decreased or absent deep tendon reflexes (3). GBS is a clinical diagnosis, but supported by investigation of cerebrospinal fluid (CSF) for an elevated protein level with normal cell count (albuminocytologic dissociation) and by nerve conduction studies. Various types of preceding infection are associated with GBS such as *Campylobacter jejuni*, Cytomegalovirus (CMV), Epstein–Barr virus (EBV), *Mycoplasma pneumoniae*, hepatitis E virus, and now ZIKV (4–6). In addition to supportive care, the treatment of GBS includes intravenous immunoglobulins (IVIg) or plasma exchange (PE).

Since the start of the ZIKV outbreak in Suriname in October 2015, clinicians in Suriname have noted a 5- to 10-fold increase in GBS incidence until March 2016 [personal communication with Dr. Harvey Yang, Neurologist, Academic Hospital Paramaribo, Suriname, March 2016 and Ref. (1)]. This case series consists of three consecutive, well-documented patients that were diagnosed with GBS in 2016 and who had a preceding infection with ZIKV.

## Clinical Description

### Case 1

Early 2016, a male in his forties was admitted to a local hospital in Paramaribo, Suriname, because of muscle weakness in the legs and paresthesias in the hands and feet. He reported development of skin rash, red eyes, and arthralgia 7 days before the start of the paresthesias and muscle weakness. Physical examination upon admission showed symmetrical muscle weakness in the arms and legs; Medical Research Council (MRC) score 4 (ranging from 0; paralysis to 5; normal strength), absent deep tendon reflexes in the legs and decreased deep tendon reflexes in the arms. During admission, the patient developed a unilateral peripheral facial nerve paralysis and areflexia in both legs and arms. A lumbar puncture was performed 3 days after hospital admission, and an elevated protein level was found (1.98 g/L, *N* < 0.5 g/L) in the CSF in absence of cells. Due to the typical presentation of symmetrical flaccid muscle weakness with decreased or absent deep tendon reflexes, an albuminocytologic dissociation in the CSF and absence of a sensory level, fever, encephalopathy, or other clinical signs of myelum involvement, the diagnosis of GBS was considered the most probable. ZIKV real-time RT-PCR (qRT-PCR) was performed locally on urine taken at day five after admission and was negative (7). A motor nerve conduction study (NCS) was performed 13 days after the start of muscle weakness. The NCS showed a decreased nerve conduction velocity (NCV) and compound muscle action potential (CMAP) and an increased distal motor latency (DML) (see Table S1 in Supplementary Material) (8). The results of the NCS were consistent with the criteria for the primary demyelinating form of GBS according to the Hadden’s criteria (9). A needle EMG was also performed and showed no abnormalities. The patient was still able to walk independently and, as symptoms remained relatively mild, was not treated with IVIg or PE. During admission, the muscle strength of the patient improved. He was discharged from the hospital after 11 days and followed-up at the neurology outpatient clinic until 12 weeks after hospital discharge, at which time he was fully recovered.

### Case 2

In February 2016, a male in his 40s was admitted to the neurology department of a hospital in Paramaribo, Suriname, because of muscle weakness in the legs, which started 7 days prior to admission. At presentation in the hospital, the patient was unable to walk without help. Two weeks before muscle weakness started he visited the hospital because of urinary retention. Acute myelitis and a cauda syndrome were excluded with an MRI-scan, which showed no abnormalities. The week before muscle weakness started he had an influenza-like syndrome and had a short episode of diplopia. At first physical examination after admission, muscle weakness was observed in predominantly the legs (MRC score 4). Areflexia in both legs and arms was noted. The patient complained of paresthesias and pain in the legs but did not have a sensory level or other signs of myelum involvement. A lumbar puncture was performed 2 days after admission, and a high protein level was found in the CSF (4.4 g/L) while the cell count was slightly increased (32.7 × 10^6^/L). A urine sample, taken at day two after admission, was tested positive for ZIKV RNA with a Ct-value of 35.7 (7). Four days after admission his muscle power improved (MCR score arms 5, legs; proximal 5, distal 4), while the areflexia in arms and legs persisted. Because of the little availability of IVIg in Suriname and because PE is not possible in Suriname, the patient did not received immunotherapy. A motor NCS, performed 15 days after the start of muscle weakness, showed severe decreased NCV and a prolonged DML in all tested nerves. The CMAP was decreased in predominantly the legs (see Table S2 in Supplementary Material). These results were consistent with the demyelinating subtype of GBS (10). At discharge, 7 days after admission, he was still unable to walk without help due to muscle weakness in the legs. Four months after hospital discharge he was able to walk a few steps without assistance, but he still could not live independently or return to work.

### Case 3

In March 2016, a male in his 60s visited a hospital in Paramaribo, Suriname, because of right-sided peripheral facial nerve paralysis since 1 day. The patient did not report having had ZIKV-like symptoms prior to admission. Neurological examination revealed absent deep tendon reflexes in the lower extremities but no muscle weakness. The patient was admitted to the neurology department for observation. At day three of admission he developed a bilateral peripheral facial nerve paralysis. Four days after admission, the patient was unable to walk or stand due to muscle weakness in the legs (MRC score 4) and areflexia in all limbs. An elevated protein level with a mild increased cell count was found in the CSF (protein 2.93 g/L, cells 15.7 × 10^6^/L) taken 5 days after admission. The progressive phase of the muscle weakness lasted for 6 days after which the patient started to recover. Motor NCS showed a decreased dCMAP and NCV and increased DML in predominantly the legs but also the arms. The tibial nerve was inexcitable in both legs (see Table S3 Supplementary Material). Features of demyelination were only found in one nerve, and as such the NCS results were classified as equivocal (9). ZIKV qRT-PCR was performed locally on urine taken at day four after admission and was negative (7). The patient received no PE or IVIg because of the limited availability of IVIg. At discharge on day 18, the patient was able to walk a small distance with assistance. The patient was followed-up at the neurology outpatient clinic until 4 months after hospital discharge, at this time he did not have residual deficits anymore.

### Laboratory Analysis

Real-time RT-PCR for ZIKV in urine was performed in the Academic Hospital Paramaribo, Suriname. Plasma, serum, and CSF samples were collected from the three patients and sent to the WHO collaborating centre for arbovirus reference and research at Erasmus Medical Centre in Rotterdam, the Netherlands for further testing. The samples were collected at days 8 (case 1), 5 and 7 (case 2), and 3 (case 3) after hospital admission.

Zika virus qRT-PCR in plasma and CSF was negative in all three patients (see Table 1) (7). A ZIKV ELISA (Euroimmun, Lübeck, Germany) was used according to the manufacturer’s recommendation to detect anti ZIKV IgM and IgG antibodies in serum samples (11). Anti ZIKV IgM ELISA was negative in all patients, while anti ZIKV IgG antibodies were present in all patients. A ZIKV neutralization test (VNT) based on a Suriname ZIKV isolate (GenBank: KU937936.1) was performed to detect ZIKV neutralizing antibodies. All three patients had neutralizing antibodies against ZIKV with titers ranging from 25 (case 1) to 256 (case 3). Serology in all patients was negative for recent infections with *C. jejuni* and showed an infection in the past with CMV (high avidity IgG anti CMV) and EBV. All serum samples tested negative for dengue virus (DENV) IgM antibodies and non-structural protein 1 (NS1) antigen and positive for DENV IgG antibodies. ELISA was used to detect the presence of IgM and IgG antibodies against GM1, GM2, GD1a, GD1b, GT1b, and GQ1b, and paired complexes of all these gangliosides in the acute phase serum of the patients (12). These anti-ganglioside antibodies were negative in all patients.

**Table 1:** **Results of diagnostic tests**.

Assay	Material	Case 1	Case 2	Case 3
ZIKV qRT-PCR	Urine	NEG	POS	NEG
	Plasma	NEG	NEG	NEG
	CSF	NEG	NEG	NEG
Anti ZIKV antibodies (ELISA)	Serum	IgM NEG	IgM NEG	IgM NEG
		IgG POS	IgG POS	IgG POS
ZIKA VNT (titer + result)	Serum	25, POS	40, POS	256, POS
			81, POS	
DENV	Serum	IgM NEG	IgM NEG	IgM NEG
		IgG POS	IgG POS	IgG POS
		NS1 NEG	NS1 NEG	NS1 NEG
*Campylobacter jejuni*	Serum	IgM NEG	IgM NEG	IgM NEG
		IgG POS	IgG POS	IgG POS
CMV	Serum	IgM NEG	IgM NEG	IgM NEG
		IgG POS	IgG POS	IgG POS
EBV	Serum	IgM NEG	IgM NEG	IgM NEG
		IgG VCA POS	IgG VCA POS	IgG VCA POS
		EBNA POS	EBNA POS	EBNA NEG

*VNT, virus neutralization test; NS1, non-structural protein 1; VCA, viral capsid antigen; EBNA, Epstein–Barr virus nuclear antigen; ZIKV, Zika virus; CMV, cytomegalovirus; EBV, Epstein–Barr virus; qRT-PCR, real-time RT-PCR*.

## Discussion

Here, we presented three patients from Suriname with acute flaccid paralysis during the height of the ZIKV outbreak in this country. Differential diagnostic considerations were GBS and acute (*Flavivirus*) myelitis. None of the patients had a sensory level, increased or pathological reflexes, fever, encephalopathy, or other symptoms suggesting an acute myelitis, and there was no marked pleocytosis in the CSF. Only motor and no sensory NCS were performed in these patients. The results of the motor NCS were consistent with GBS in all three patients. Furthermore, all patients had progressive symmetrical muscle weakness with areflexia, a monophasic disease course, an albuminocytologic dissociation in the CSF, and absence of an identified alternative diagnosis for muscle weakness. All patients thus scored the highest level for diagnostic certainty for GBS (level I) according to the diagnostic criteria for GBS of the Brighton collaboration and were therefore diagnosed with GBS (13). No anti-ganglioside antibodies were found in the serum of these patients, but as anti-ganglioside antibodies are only found in an estimated 50% of GBS cases, their absence does not rule out the diagnosis GBS (14).

Typically, muscle weakness in GBS occurs 1–2 weeks after the preceding infection that triggers GBS (3). This delay makes it difficult to identify the infection that could have triggered the onset of GBS based on molecular methods, as the infection may have been cleared by the time of onset of the GBS symptoms. Especially in ZIKV suspected GBS cases, it is difficult to determine if the GBS is indeed caused by a recent ZIKV infection. This is due to a relatively small diagnostic time window of qRT-PCR for ZIKV in EDTA-plasma (3–5 days), to a lesser extent in urine (up to 30 days) and semen (up to 92 days), although the sensitivity of testing at these later time points remains to be validated (15–18). Confirmation of an infection based on serology will have a broader window of detection. However, in case of past *Flavi*virus exposure, ZIKV IgM response may be delayed, limited, or even absent, further complicating etiological diagnosis (19). The diagnostic work-up presented for this case series reflects these challenges of identifying possible triggers for the onset of GBS in these patients.

Definitive evidence for recent ZIKV infection was obtained in one patient who tested positive by qRT-PCR for ZIKV in the urine. In cases 1 and 3, it is possible that the diagnostic window for reliable ZIKV RNA detection had already expired once the GBS symptoms occurred and the samples were collected. No IgM antibodies to ZIKV were detected, including the patient with confirmed ZIKV shedding by RT-PCR, but all three patients had neutralizing antibodies indicating a past ZIKV infection. The absence of IgM antibodies is remarkable, although the assays that currently are available have demonstrated short-lived IgM responses and as mentioned above, IgM response can be absent in case of prior *Flavivirus* exposure (e.g., DENV in these patients) (19). A recent infection with the most prevalent preceding infections of GBS—*C. jejuni*, CMV, and EBV—were excluded in the patients, which makes it more plausible that the GBS was triggered by a recent ZIKV infection.

The exact pathophysiological mechanism behind ZIKV-associated GBS remains to be elucidated, but antibody-dependent enhancement (ADE) of ZIKV infection might play a role. It has recently been described that DENV antibodies that are able to bind ZIKV, but cannot neutralize ZIKV, can promote ADE that can result in greater ZIKV replication (20). The three patients in this case series all had IgG antibodies against DENV. It remains to be seen if the combined effects of past and recent *Flavivirus* exposures plays a role in the pathogenesis of ZIKV-associated GBS.

## Conclusion

Taken together, we can conclude that in the three presented cases, the relation between ZIKV infection and GBS was confirmed in one patient, and was plausible in the two others, given the increased GBS incidence during the ZIKV outbreak in Suriname, the ruling out of most prevalent preceding infections of GBS and the presence of ZIKV neutralizing antibodies in these patients. For definitive proof of this association, case–control studies are needed, using stringent and standardized diagnostic criteria, both for GBS and for the laboratory diagnosis.

## Ethics Statement

This study was approved by the Ministry of Health of Suriname. All participants signed informed consent prior to participation in this study.

## Author Contributions

TL, BJ, CR, MK, EG, CG, and SV wrote the manuscript. MB, HY, and HA cared for the patients. TL, LD, TK, HY, and MB collected the data. JC, JR, and SP were responsible for the molecular diagnostic tests. EB, RM, CG, and CR were responsible for the serological tests. All authors reviewed and approved the final version of the manuscript.

## Conflict of Interest Statement

The authors declare that the research was conducted in the absence of any commercial or financial relationships that could be construed as a potential conflict of interest. The reviewer VG and the handling Editor declared their shared affiliation, and the handling Editor states that the process nevertheless met the standards of a fair and objective review.
