# Supplementary material for: Zika Virus Infection and Guillain–Barré Syndrome in Three Patients from Suriname
Source: Front Neurol. 2016 Dec 22;7:233. doi: 10.3389/fneur.2016.00233 (PMC5177614; doi:10.3389/fneur.2016.00233)
Supplement: Supplementary file 1 [file Table_1.docx]

**SUPPLEMENTARY TABLE 1 | Motor nerve conduction study results from case 1.**

| **Nerve** | **DML (ms)** | **dCMAP (mV)** | **NCV (m/s)** |
| --- | --- | --- | --- |
| Left ulnar | 4.22 (<4.5) | 2.54 (>7) | 32.41 (>49) |
| Right ulnar | 6.25 (<4.5) | 0.15 (>7) | 67.07 (>49) |
| Left median | 7.73 (<4.4) | 3.33 (>4) | 49.04 (>49) |
| Right median | 7.03 (<4.4) | 2.52 (>4) | 49.89 (>49) |
| Left peroneal | NP | NP | NP |
| Right peroneal | 6.56 (<6.5) | 2.55 (>2) | 25.13 (>44) |
| Left tibial | 8.52 (<5.8) | 2.07 (>4) | 27.38 (>41) |
| Right tibial | NP | NP | NP |

*Normal adult values for NCS according to (8) are presented between brackets. DML, distal motor latency; dCMAP, distal compound muscle action potential; NCV, nerve conduction velocity; NP, not performed*
