# Supplementary material for: Zika Virus Infection and Guillain–Barré Syndrome in Three Patients from Suriname
Source: Front Neurol. 2016 Dec 22;7:233. doi: 10.3389/fneur.2016.00233 (PMC5177614; doi:10.3389/fneur.2016.00233)
Supplement: Supplementary file 2 [file Table_2.docx]

**SUPPLEMENTARY TABLE 2 | Motor nerve conduction study results from case 2.**

| **Nerve** | **DML (ms)** | **dCMAP (mV)** | **NCV (m/s)** |
| --- | --- | --- | --- |
| Left ulnar | 6.33 (<4.5) | 4.48 (>7) | 27.0 (>49) |
| Right ulnar | 5.43 (<4.5) | 4.78 (>7) | 20.3 (>49) |
| Left median | 10.1 (<4.4) | 4.50 (>4) | 21.8 (>49) |
| Right median | 8.64 (<4.4) | 4.97 (>4) | 17.8 (>49) |
| Left peroneal | 12.4 (<6.5) | 0.54 (>2) | 12.4 (>44) |
| Right peroneal | 6.56 (<6.5) | 0.79 (>2) | 10.6 (>44) |
| Left tibial | 8.52 (<5.8) | 1.85 (>4) | 14.7 (>41) |
| Right tibial | 15.8 (<5.8) | 1.51 (>4) | 15.8 (>41) |

*Normal adult values for NCS according to (8) are presented between brackets. DML, distal motor latency; dCMAP, distal compound muscle action potential; NCV, nerve conduction velocity; NP, not performed*
