# Supplementary material for: Zika Virus Infection and Guillain–Barré Syndrome in Three Patients from Suriname
Source: Front Neurol. 2016 Dec 22;7:233. doi: 10.3389/fneur.2016.00233 (PMC5177614; doi:10.3389/fneur.2016.00233)
Supplement: Supplementary file 3 [file Table_3.docx]

**SUPPLEMENTARY TABLE 3 | Motor nerve conduction study results from case 3.**

| **Nerve** | **DML (ms)** | **dCMAP (mV)** | **NCV (m/s)** |
| --- | --- | --- | --- |
| Left ulnar | NP | NP | NP |
| Right ulnar | NP | NP | NP |
| Left median | 4.83(<4.4) | 2.32 (>4) | 46.5 (>49) |
| Right median | 4.71 (<4.4) | 7.20 (>4) | 41.7 (>49) |
| Left peroneal | 7.83 (<6.5) | 0.12 (>2) | 34.1 (>44) |
| Right peroneal | 7.44 (<6.5) | 0.21 (>2) | 30.0 (>44) |
| Left tibial | Inexcitable | Inexcitable | Inexcitable |
| Right tibial | Inexcitable | Inexcitable | Inexcitable |

*Normal adult values for NCS according to (8) are presented between brackets. DML, distal motor latency; dCMAP, distal compound muscle action potential; NCV, nerve conduction velocity; NP, not performed*
